# An item bank to measure health-related quality of life among young children (0-5-years-old) affected by respiratory illnesses – expert stakeholders and end-users from the Western Cape, South Africa

**DOI:** 10.1186/s12955-024-02308-0

**Published:** 2024-10-29

**Authors:** Michaile Gizelle Anthony, Margaret Van Niekerk, Anneke Catharina Hesseling, Graeme Hoddinott, Marieke Margreet van der Zalm

**Affiliations:** 1 Desmond Tutu TB Centre, Department of Paediatrics and Child Health, Faculty of Medicine and Health Sciences, Stellenbosch University, Cape Town, South Africa; 2 School of Public Health, Faculty of Medicine and Health, The University of Sydney, Sydney, Australia

**Keywords:** Health-related quality of life, Respiratory illnesses, Delphi study, Cognitive interview, Young children

## Abstract

**Background:**

Health-related quality of life (HRQoL) is a crucial patient-centred outcome for developing policy. However, there is a lack of appropriate HRQoL measures for young children (0-5-years), who are particularly vulnerable to respiratory illnesses like pulmonary tuberculosis (PTB) and other respiratory infections, especially in low- and middle-income countries (LMICs). We aimed to develop a disease-specific HRQoL item bank for young children with acute and chronic respiratory illnesses.

**Methods:**

An exploratory sequential design with three phases was used to develop a HRQoL item bank. The content validity of the item bank was evaluated by local and international experts specialising in HRQoL and child health. The group included paediatric pulmonologists, researchers with expertise in respiratory illnesses, and experts in scale development. Cognitive interviews with 37 caregivers of children with TB, pneumonia, adenovirus respiratory infection, other lower respiratory tract infections, reactive airway disease, and protracted bronchitis in Cape Town, South Africa, and consultations with 22 stakeholders were conducted for final revisions. The item bank was progressively refined at each phase of the study.

**Findings:**

The Delphi experts recommended dividing the item bank into two age groups (0-2-years and 3-5-years) and using a 5-point Likert scale. Overall, 41 items (42%) met the predetermined > 70% threshold for inclusion in the item bank. Cognitive interviews confirmed that the domains were relevant. Minor modifications were made to five items in cohort 1 (0-2-years) and seven in cohort 2 (3-5-years), with 8 items (13%) and 14 items (22%) excluded. Phase 3 consultations emphasised the importance of including all seven domains and expanding the items to cover early childhood development, play, social interactions, and care routines. The final item bank includes versions for both age groups and incorporates these refinements.

**Conclusion:**

An item bank was developed as a first step to develop a comprehensive disease-specific HRQoL tool for young children with respiratory illnesses in an LMIC. Input from caregivers and content experts was crucial in creating two HRQoL item banks tailored to the developmental differences between 0 and 2 and 3-5-year age groups. Their contributions ensured the tool effectively captures age-appropriate aspects of HRQoL. Future studies should focus on assessing the validity and reliability of these item banks.

**Supplementary Information:**

The online version contains supplementary material available at 10.1186/s12955-024-02308-0.

## Introduction

Health-related quality of life (HRQoL) is crucial for young children < 5-years old as it encompasses various aspects such as physical health, emotional well-being, and social interactions, all essential for their overall development and well-being [1]. Health-related quality of life measures allow healthcare professionals to evaluate treatment effectiveness, tailor interventions, and improve patient care. Health-related quality of life data can inform health policy and resource allocation, enhancing the HRQoL for individuals with health conditions [1, 2].

Respiratory illnesses pose a significant global health threat, particularly for young children. Approximately nine million children (0-17-years-old) die annually, with the highest burden among children *≤* 5-years. 90% of these deaths occur in low-middle-income countries (LMICs) [3–5]. Globally, lower respiratory tract infections (LRTIs) are the leading cause of hospitalisation among the paediatric population, with approximately 11.9 million paediatric patients (*≤* 5-years-old) being hospitalised in 2019 [6–11]. Viral infections account for approximately 90% of all LRTIs among paediatric patients [11].

Pulmonary tuberculosis (PTB) in young children, especially those ≤ 5-years-old, often resembles other childhood illnesses such as LRTIs and is more challenging to diagnose in children living with human immunodeficiency virus (HIV) or in malnourished children [12, 13]. In 2022, an estimated 1.3 million children aged 0-14-years developed TB. However, only half of these children accessed care, with the diagnostic gap being the greatest among children *≤* 5-years [14]. The high burden of TB in this age group necessitates focused attention to adequately address its unique challenges and implications for child health and well-being. Despite the high burden of the disease, limited data are available globally on the morbidity of childhood TB and even less on the psychosocial consequences of TB [15, 16]. Young children affected by respiratory illnesses face unique developmental and health-related challenges that are not adequately captured by tools designed for older populations. This also includes the impact of the family or care system around them. Additionally, no disease-specific measure for this age group, exists in an LMIC context, highlighting the need for a tailored approach to assess their disease-specific HRQoL more accurately.

Research shows that although generic (TANDI & EQ-5D-Y) and disease-specific (Cystic fibrosis revised versions (CFQ-R) HRQoL measures are available for children, these measures have limitations for younger children aged *≤* 5-years, as they lack a holistic picture of these young children [17–19]. The TANDI, which was developed in South Africa for children < 3-years, and the EQ-5D-Y, which included a South African perspective during its development, represent notable exceptions. Despite these contributions, many other widely used tools, such as CHU-9D, PedsQL, and PROMIS, were not designed with younger children or LMIC contexts in mind, highlighting the need for further refinement and development of HRQoL measures that are sensitive to these populations’ unique needs. Studies have shown that cultural adaptation should be included during the development of measures and that simple translation and modifications of item wording are insufficient to ensure the conceptual equivalence of tools in different contexts [20, 21]. A study conducted among children *≤* 5-years with respiratory illnesses in an LMIC found that caregivers’ perceptions of HRQoL were strongly influenced by the socio-economic context [22]. Caregivers associated HRQoL with the availability of basic resources, such as food, shelter, and clothing [22]. Young children face the highest burden of TB and other respiratory illnesses [23]. Globally, child mortality rates for those *≤* 5-years are significant, with particularly high rates in LMICs [23]. Additionally, children who were affected by TB or other respiratory illnesses often experience ongoing morbidity, underscoring the need for a measure to monitor their HRQoL over time [15]. We aimed to develop a comprehensive HRQoL item bank for children *≤* 5-years in an LMIC affected by respiratory illnesses through a three-step process, including a Delphi consensus study with experts, cognitive interviews, and stakeholder consultations. Specifically, we sought to generate a consensus on domains, items within domains, response options, and overall logic for version 1.0 of such an item bank.

## Methods

### Study design

**Fig. 1:**
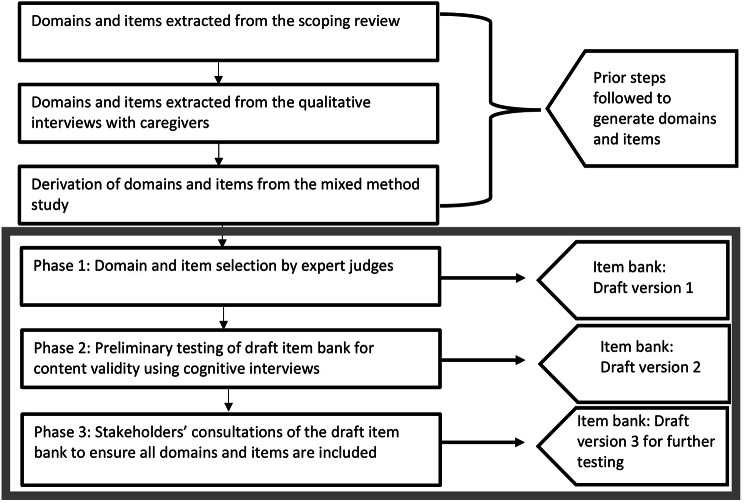
Multistep process of developing an item bank for an HRQoL measure

An exploratory sequential design with three phases to develop a preliminary HRQoL item bank [24]. Items and domains were generated from three sources: (1) a Delphi survey with experts in the field; (2) cognitive interviews with caregivers of children affected by respiratory illnesses, including TB; and (3) stakeholder consultations (Fig. 1).

### Phase 1 – Delphi study

#### Sampling and recruitment

**Fig. 2:**
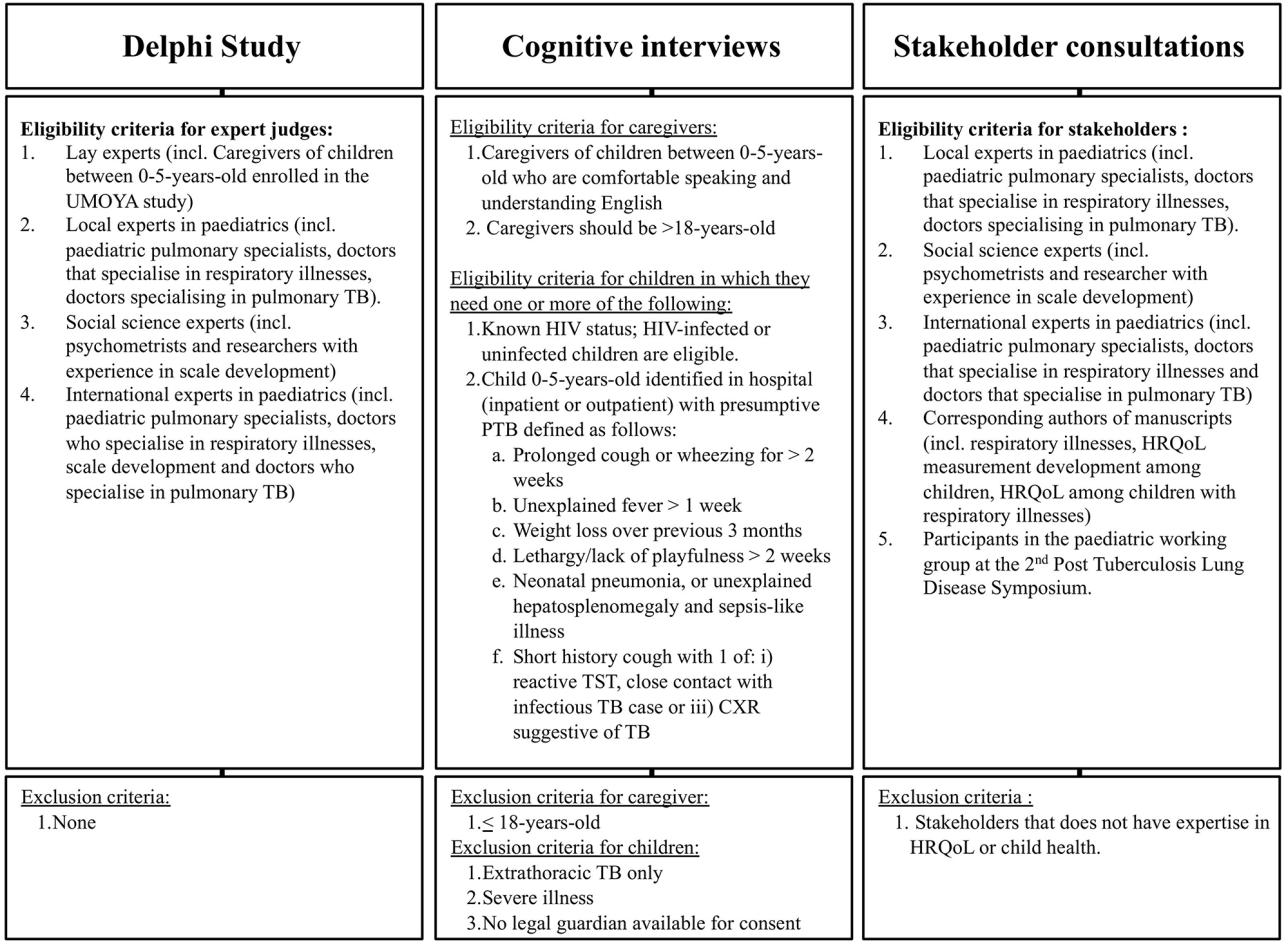
Eligibility of study participants across the three phases

A Delphi survey was conducted to gather expert opinions across seven previously identified domains related to HRQoL (Fig. 2). These domains—physical health, emotional well-being, psychological health, social well-being, feeling loved, early child development, and routine—were identified through a scoping review (under review) of available HRQoL measures and qualitative interviews with caregivers of young children affected by respiratory illnesses. In total 18 experts were included, with 12 (67%) from LMICs and 3 (16%) from high-income countries (HICs), all of whom have conducted extensive research and clinical work in high-burden LMIC settings. Additionally, 3 lay experts (16%) were included, comprising caregivers of children with respiratory illnesses in South Africa. Recruitment involved personalised email invitations to identified experts sourced from universities, relevant university departmental websites, authors of pertinent manuscripts, and recommendations from included experts. Lay experts, including caregivers of children presenting with presumptive PTB, were recruited from the Umoya study. The recruitment followed the same eligibility criteria as the cognitive interviews.

#### Data collection activities

The expert participants were asked to score 85 statements using a 7-point Likert scale: not relevant, not relevant to the age group, not relevant to HRQoL, somewhat relevant, quite relevant, highly relevant, and essential. These statements were derived from a literature review and organised into five sections (including domains, items within domains, symptoms, recall period, and additional suggestions). We sent out email reminders two weeks after the initial contact to keep participants engaged. The data were collected by the first author (MGA), a graduate researcher with masters-level degree in psychology.

#### Data analysis

The consensus criterion was set at *≥* 70%, as established in prior Delphi studies [25, 26]. The “*≥*70%” threshold refers to the proportion of experts who rated an item as relevant or irrelevant on a 7-point Likert scale. Items were considered relevant if more than 70% of experts rated them as “quite relevant,” “highly relevant,” or “essential.” Conversely, items were considered irrelevant if more than 70% of experts rated them as “not relevant,” “not relevant to age group,” “not relevant to HRQoL,” or “somewhat relevant.” For an item to be included or excluded, it had to meet this *≥* 70% agreement criterion for relevance or irrelevance. The data analysis was performed using IBM SPSS Statistics (Version 28) [27]. The study utilised Research Electronic Data Capture (REDCap) (Version 14.5.2) for survey administration. REDCap is a secure web application used for building and managing online surveys and databases [28, 29]. The data were analysed by the first author (MGA), with senior authors, GH and MVDZ providing analytical input to ensure robustness in the analysis.

### Phase 2 – cognitive interviews

#### Setting

Cognitive interviews were conducted at Tygerberg Hospital (TBH), located in Tygerberg, Cape Metropolitan District (CMD), Western Cape Province, South Africa. The data collection was part of a prospective observational TB diagnostic cohort study, Umoya, which involved children aged 0–13-years (median age 2-years) presenting with presumptive PTB [30]. The TB incidence among young children in Cape Town is ~ 511 per 100,000 [31]. Many communities exhibit high levels of poverty, unemployment, overcrowding, and drug abuse [32]. Caregivers’ perceptions of HRQoL in LMIC are fundamentally shaped by their socioeconomic context, emphasising the availability of basic resources such as food, shelter, and clothing [22]. When basic needs are not met, other aspects of HRQoL, such as healthcare access, become less prioritised [22, 33]. Consequently, HRQoL is experienced differently based on socioeconomic conditions.

#### Sampling and recruitment

We enrolled a purposive subsample of 37 caregivers of 40 children (*≤* 5 years-old), who were part of the Umoya study and had presumptive PTB (Fig. 2). Approximately, ± 40% of the children were started on TB treatment, both microbiologically confirmed and clinically diagnosed TB, while ± 60% of children were found to not have TB and are considered symptomatic controls, with alternative diagnoses. In this study, 19 children were diagnosed with PTB, and 21 were diagnosed with other respiratory illnesses, including pneumonia, adenovirus respiratory infection, other lower respiratory tract infections, reactive airway disease, and protracted bronchitis. Participants were selected based on differences in child age, sex, and diagnosis. The sample size was determined by theoretical saturation, stopping recruitment when no new themes emerged. Participants were approached during routine study visits, where they received detailed information and were asked to participate.

#### Data collection activities

Semi-structured interviews using a semi-structured guide developed to gather specific insights from caregivers were used. Participants received the item bank before the start of the interviews. The researcher reviewed each item with them, allowing them time to reflect on their child’s experiences while completing the questionnaire. During the interviews, each question was discussed in detail, with caregivers encouraged to ask questions and express concerns, ensuring thorough and thoughtful feedback. The guide focused on the relevance and length of the item bank, ensuring it effectively captured necessary information such as the item bank’s face validity, format, instructions, and the perceived relevance of items to HRQoL. The interviews were conducted in person during participants’ routine study visits if they expressed interest in participating. Each interview, conducted in English, lasted for 20–60 min, and was audio-recorded. At the end of the data collection day, the first author (MGA) wrote semi-structured case summaries. These summaries were reviewed by the senior authors (GH & MvdZ) to ensure analytic rigour.

#### Data analysis

The questionnaire responses were analysed using IBM SPSS Statistics (Version 28) [27]. Survey administration was conducted through REDCap (Version 14.5.2) [28]. Case descriptive analysis, including verbatim transcription and translation of relevant interview sections, to identify key experiences from each participant. By comparing and sharing these experiences, we organised common and divergent themes. Although all interviews were conducted in English, some caregivers occasionally used Afrikaans words when they could not find the right English term. These Afrikaans words were translated into English during data analysis to ensure accuracy and consistency. Caregivers’ responses were collated into a table, grouping them by categories such as items needing modification. Most responses echoed the quantitative data collected using the item bank during the cognitive interviews without adding new insights, leading to a decision against further analysis.

### Phase 3 – stakeholder consultations

#### Sampling and recruitment

Twenty-two experts, with 2 (9%) from low-income countries, 16 (73%) from LMIC, and 4 (18%) from HICs, all of whom have conducted extensive research and clinical work in high-burden LMIC settings (Fig. 2). Newly included corresponding authors were included through a multi-faceted approach. In addition to inviting previous Delphi survey participants, the search was expanded to include attendees of the 2nd international Post-TB symposium [34]. To ensure a comprehensive participant pool, an extensive search across the first 10 pages of Google, targeted researchers with expertise in HRQoL, respiratory illnesses, TB, and PTLD. Their relevance to this study invited additional participants who had not been part of the Delphi study, thereby broadening engagement for this phase.

#### Data collection activities

A REDCap-hosted survey was emailed to participants, featuring yes/no questions regarding the importance and sufficiency of HRQoL domains, free-text responses whenever “no” was selected, and a final free-text field for additional suggestions.

#### Data analysis

Data analysis involved organising the responses with descriptive statistics, summarising frequencies and proportions using IBM SPSS Statistics (Version 28) [25]. Stakeholder responses (yes/no) were compiled into a table and found that they generally mirrored the quantitative data collected from the Delphi study and cognitive interviews with caregivers without offering additional insights, and thus decided against further analysis. The first author (MGA) conducted the analysis, which was reviewed by the senior authors (GH & MvdZ) to ensure analytic rigour. Throughout the study, participants were engaged to verify item comprehensiveness and relevance. Delphi experts were thus provided with another opportunity to review and provide feedback on the item bank to ensure accuracy and inclusivity.

## Findings

### Phase 1 Delphi study: domain and item review

Experts deemed the seven domains—physical health, emotional health, psychological health, social well-being, getting love/feeling love, early childhood development, and routine—as relevant. However, relevance ratings for domains specific to 0-1-year ranged from 67 to 89%, with the “social well-being” (67%) and “routine” (67%) domains rated as irrelevant for this age group. Despite this, these domains were retained based on the expert panel’s recommendation to create two separate versions of the item bank: one for 0-2-years (cohort 1) and another for 3-5-years (cohort 2) (Table 1).

**Table 1:** Illustration of both the overall relevance of domains and the specific items within those domains by Delphi experts in round 1

	# of experts	# of domains/items in round	Relevance range^a^	# of domains/items rated < 70%^b^
Domains				
*0-1-years-old*	18	7	67 − 89%	2 domains
*2-3-years-old*	18	7	89 − 94%	0 domains
*4-5-years-old*	18	7	83 − 100%	0 domains
**HRQoL items**	18	85	39-100%	41 Items

# = number of

^a^ Relevance range = number of participants who rated the domains and items as “Quite relevant”, “Highly relevant” and “Essential”

^b^ Domains and items rated as relevant by less than 70% of experts required revision

Overall, 41 items (42%) met the predetermined *≥* 70% threshold for inclusion in the item bank (Table 2). Following expert recommendations, the item bank was divided into two cohorts. Eight items for cohort 1 and four items for cohort 2 were excluded before the second round of the Delphi. These items were deemed irrelevant for children *≤* 5-years. During the second round, inclusion rates varied from 25 to 81% for cohort 1 and 50–93% for cohort 2. In total, 69 items (81%) were retained for Cohort 1 and 80 items (94%) for Cohort 2. Further details are provided in Additional File 1. Experts recommended excluding items for reasons including developmental and/or age-inappropriate, vague, and unclear, and overlapping with multiple domains.

**Table 2:** Experts’ responses on the relevance of the 85 HRQoL items per domain

		All participants (*n* = 18)	
		*N*	%
Physical health	Categories		
My child was able to keep up when playing with his/her friends/siblings	Relevant	17	**94.4**
	Not relevant	1	5.6
My child had fun playing	Relevant	16	**88.9**
	Not relevant	2	11.1
My child had trouble sleeping because of his/her symptoms (incl. cough, pain, fever etc.)	Relevant	18	**100**
	Not relevant	0	0
My child felt tired	Relevant	15	**83.3**
	Not relevant	3	16.7
My child refused to eat	Relevant	15	**83.3**
	Not relevant	3	16.7
My child was full of energy	Relevant	16	**88.9**
	Not relevant	2	11.1
My child felt strong	Relevant	11	61.1
	Not relevant	7	38.9
My child did not feel like playing	Relevant	13	**72.2**
	Not relevant	5	27.8
My child was active	Relevant	16	**88.9**
	Not relevant	2	11.1
My child felt sick	Relevant	16	**88.9**
	Not relevant	2	11.1
My child had less fun than usual	Relevant	11	61.1
	Not relevant	7	38.9
My child’s health is good	Relevant	14	**77.8**
	Not relevant	4	22.2
My child had pain	Relevant	17	**94.4**
	Not relevant	1	5.6
My child had trouble gaining weight	Relevant	14	**77.8**
	Not relevant	4	22.2
My child’s cough woke him/her up	Relevant	14	**77.8**
	Not relevant	4	22.2
My child had feeding problems	Relevant	12	66.7
	Not relevant	6	33.3
***Emotional health***			
My child was crying when he/she is sick	Relevant	14	**77.8**
	Not relevant	4	22.2
My child felt as if he/she was being punished	Relevant	7	38.9
	Not relevant	11	61.1
My child thinks that his/her friends with make fun of him/her	Relevant	7	38.9
	Not relevant	11	61.1
My child thinks his/her friends with make fun of him/her because of the way he/she looks	Relevant	12	66.7
	Not relevant	6	33.3
My child felt scared	Relevant	12	66.7
	Not relevant	6	33.3
My child felt unhappy	Relevant	14	**77.8**
	Not relevant	4	22.2
My child felt happy	Relevant	14	**77.8**
	Not relevant	4	22.2
My child was anxious	Relevant	13	**72.2**
	Not relevant	5	27.8
My child was angry	Relevant	11	61.1
	Not relevant	7	38.9
My child was sad	Relevant	12	66.7
	Not relevant	6	33.3
My child felt lonely	Relevant	9	50
	Not relevant	9	50
My child felt excited	Relevant	8	44.4
	Not relevant	10	56.6
My child was irritable	Relevant	14	**77.8**
	Not relevant	4	22.2
My child was short-tempered	Relevant	11	61.1
	Not relevant	7	38.9
My child was fussy	Relevant	11	61.1
	Not relevant	7	38.9
My child’s cry was inconsolable	Relevant	14	**77.8**
	Not relevant	4	22.2
***Psychological health***			
My child felt uncomfortable when he/she was sick	Relevant	14	**77.8**
	Not relevant	4	22.2
My child was nagging more when he/she was sick	Relevant	12	66.7
	Not relevant	6	33.3
My child was shy	Relevant	8	44.4
	Not relevant	10	56.6
My child felt proud of himself/herself	Relevant	8	44.4
	Not relevant	10	56.6
My child felt that he/she was physically different to other children his/her age	Relevant	11	61.1
	Not relevant	7	38.9
My child felt jealous about the way other girls and boys look	Relevant	4	22.2
	Not relevant	14	**77.8**
My child is more withdrawn	Relevant	13	**72.2**
	Not relevant	5	27.8
My child is moody	Relevant	13	**72.2**
	Not relevant	5	27.8
My child was defiant	Relevant	11	61.1
	Not relevant	7	38.9
My child was restless	Relevant	12	66.7
	Not relevant	6	33.3
My child was fidgety	Relevant	11	61.1
	Not relevant	7	38.9
My child was needy	Relevant	11	61.1
	Not relevant	7	38.9
My child was clingy	Relevant	14	**77.8**
	Not relevant	4	22.2
My child had nightmares	Relevant	10	55.6
	Not relevant	8	44.4
My child started wetting his/her pants	Relevant	11	61.1
	Not relevant	7	38.9
My child started wetting his/her bed	Relevant	11	61.1
	Not relevant	7	38.9
***Social well-being***			
My child’s friends left him/her out when they did things (e.g., playing) together	Relevant	11	61.1
	Not relevant	7	38.9
My child’s brothers, sisters or cousins left him/her out when they did things (e.g., playing) together	Relevant	11	61.1
	Not relevant	7	38.9
My child was felt confident with other children	Relevant	10	55.6
	Not relevant	8	44.4
My child felt scared around other children	Relevant	11	61.1
	Not relevant	7	38.9
My child’s friend helped him/her	Relevant	8	55.6
	Not relevant	10	44.4
My child helped her friends	Relevant	9	50
	Not relevant	9	50
My child got on well with his/her brothers, sisters, cousins	Relevant	12	66.7
	Not relevant	6	33.3
My child got on well with his/her friends	Relevant	9	50
	Not relevant	9	50
My child had difficulty getting along with others	Relevant	13	**72.2**
	Not relevant	5	27.8
My child’s illness/disease caused stress in the family	Relevant	14	**77.8**
	Not relevant	4	22.2
My child was liked by other children	Relevant	13	**72.2**
	Not relevant	5	27.8
***Getting love/ Feeling loved***			
My community came over to helped care for my child when he/she was sick	Relevant	11	61.1
	Not relevant	7	38.9
My child got on well with his/her family	Relevant	11	61.1
	Not relevant	7	38.9
My child felt unloved by his/her family	Relevant	12	66.7
	Not relevant	6	33.3
My child felt loved by his/her family (including brothers, sisters, cousins, mother, father, extended family)	Relevant	12	66.7
	Not relevant	6	33.3
My family comforted my child when he/she was crying, not feeling sick.	Relevant	15	**83.3**
	Not relevant	3	16.7
My family helped care for my child when he/she was feeling sick.	Relevant	15	**83.3**
	Not relevant	3	16.7
My family did not support my child when he/she was feeling sick.	Relevant	12	66.7
	Not relevant	6	33.3
My child felt loved by his/her community/neighbours.	Relevant	11	61.1
	Not relevant	7	38.9
***Early child development***			
My child communicates in an age-appropriate way with his/her friends (babbling, smiling, touching)	Relevant	16	**88.9**
	Not relevant	2	11.1
My child can talk in complete sentences	Relevant	11	61.1
	Not relevant	7	38.9
My child can follow simple commands	Relevant	12	66.7
	Not relevant	6	33.3
My child was not able to things other children his/her age can do.	Relevant	15	**83.3**
	Not relevant	3	16.7
My child can imitate others	Relevant	11	61.1
	Not relevant	7	38.9
My child is behind other child his/her age in day care	Relevant	15	**83.3**
	Not relevant	3	16.7
My child communicates in an age-appropriate way with his/her family	Relevant	15	**83.3**
	Not relevant	3	16.7
My child can keep up with other children his/her age at day care/creche	Relevant	16	**88.9**
	Not relevant	2	11.1
My child needed extra support at day care/creche	Relevant	14	**77.8**
	Not relevant	4	22.2
My child enjoyed day care/ creche	Relevant	16	**88.9**
	Not relevant	2	11.1
***Routine***			
My child was not eating enough because he/she was not feeling well.	Relevant	16	**88.9**
	Not relevant	2	11.1
My family had to change our routine when my child was sick	Relevant	17	**94.4**
	Not relevant	1	5.6
My child had difficulty staying asleep because of his/her symptoms	Relevant	14	**77.8**
	Not relevant	4	22.2
My child’s sleeping patterns were irregular/changed/disturbed	Relevant	16	**88.9**
	Not relevant	2	11.1
My child missed out on creche/day care because he/she was sick	Relevant	15	**83.3**
	Not relevant	3	16.7
My child stuck to his/her sleeping routine	Relevant	13	**72.2**
	Not relevant	5	27.8
My child stuck to his/her eating routine	Relevant	12	66.7
	Not relevant	6	33.3
My child’s normal routine was changed when he/she was sick	Relevant	12	66.7
	Not relevant	6	33.3

Note Table 2 provides a more detailed breakdown, showing that while most domains were considered relevant, individual items within those domains (not boldfaced) were assessed and found to need revision

### Phase 2 – cognitive interviews

Overall, participants in cohort 1 of the cognitive interviews found all items in the physical, getting love/feeling love, and routine domains to be sensible and well-aligned with their understanding of their child’s HRQoL. However, caregivers recommended excluding 8 items (13%) across the emotional, psychological, social, and early development domains, deeming them irrelevant for children aged 0-2-years. In contrast, in cohort 2, only the routine domain had 100% inclusion of all items. Caregivers in this cohort suggested excluding 14 items (22%) across the physical, emotional, psychological, social, getting love/feeling love, and early development domains, considering them unsuitable for children aged 3-5-years. Generally, items within the domains were perceived as relevant and intuitive. Additional File 2. Minor modifications were made to 4 items (6%) in cohort 1 and 7 items (11%) in cohort 2 [Additional file 1]. Participants in both cohorts recommended including two additional items: “My child felt worried” and “My family did not support my child when he/she was sick.” Caregivers expressed a slight preference for a 3-point over a 5-point Likert scale and favoured a recall period of “in the preceding month.

### Phase 3: stakeholder consultations

Overall, more than 70% of experts deemed the domains and items relevant for children aged 0-5-years [Additional File 4]. In response to stakeholder feedback, we incorporated an additional 47 items across both cohorts. This expansion addressed critical developmental considerations and the impacts of associated illnesses, including play, growth, attachment, social isolation, effort tolerance, community integration, anxiety, and self-confidence. Special attention was given to the child’s well-being during disease episodes, focusing on physical growth, treatment burden, and attachment dynamics. The item bank was therefore broadened to include these key aspects. Stakeholder recommendations also led to the inclusion of items in the social well-being domain, reflecting the child’s interactions with siblings and peers, responsiveness to caregivers, and social skills (e.g., getting along with other children, ability to make friends). Additionally, the routine domain was updated to include information about the individuals providing care to the child. Based on stakeholder recommendations, further modifications were made, including revisions to 26 items and the exclusion of six items for cohort 1. For cohort 2, stakeholders recommended revisions to 30 items and the exclusion of six items. Additional details are provided in [Additional File 1].

## Discussion

This study developed an item bank to create a disease-specific HRQoL measure for young children (0-5-years) affected by respiratory illnesses including TB in an LMIC. The developed item bank is divided into two age-specific versions: for children 0-2-years it includes seven domains: physical health (32 items), emotional health (7 items), behavioural expression (18 items), social well-being (12 items), feeling love (7 items), early child development (21 items), and routine (9 items). For children aged 3-5-years, the item bank also comprises seven domains: physical health (29 items), emotional health (15 items), psychological health (31 items), social well-being (17 items), feeling love (7 items), preschool readiness (22 items), and routine (10 items). Both versions utilise a 5-point Likert scale with a recall period of ‘in the last month’ where relevant.

This item bank is a first step to address significant gaps in existing HRQoL measures. Existing HRQoL measures, such as the EQ-5D-Y and TANDI, although developed and validated in LMICs with high disease burdens [35–38], do not offer a disease-specific measure for young children with respiratory illnesses, including TB [22]. Cultural adaption is vital for HRQoL measures in diverse settings; mere translation is insufficient for ensuring conceptual equivalence across contexts with different socioeconomic conditions and disease burdens [20].

Previous research among children aged 0-5-years affected by respiratory illnesses emphasised the need to adapt commonly measured HRQoL domains—physical health, emotional health, psychological health, social well-being, and schooling—for young children in LMICs [22]. This study builds on this by creating two versions of the item bank that account for the developmental differences between children 0-2-years and 3-5-years. The inclusion of seven domains—physical, emotional, behavioural expression/psychological, social well-being, feeling love, early development/preschool readiness, and routine—reflects a comprehensive approach to assessing HRQoL, considering critical developmental factors like play, growth, and social interaction.

These findings mark a significant step toward a more holistic and comprehensive HRQoL measure for children < 5-years-old affected by respiratory illnesses, including TB. However, further qualitative data from various LMIC settings are needed to improve our understanding of HRQoL in young children. The next phase involves administering the item banks to the target population, conducting item analysis to evaluate each item’s effectiveness, and performing exploratory factor analysis (EFA) to assess the factor structure and refine the item bank. Validation studies across multiple sites will be crucial to ensuring the robustness and generalisability of the measure.

The strengths of this process include the inclusion of diverse perspectives; an iterative developmental approach, and the focus on young children in an LMIC context. The Delphi study and stakeholder consultations contributed to a nuanced understanding of HRQoL, while cognitive interviews with caregivers ensured the relevance and clarity of the items within the local setting. However, cognitive interviews were only conducted in Cape Town, South Africa, and exclusively in English, limiting the cultural and linguistic variability of the item bank across South Africa’s 11 official languages. The home languages of the participants included English, Afrikaans, and IsiXhosa. Future work should include the translation and explorations of these items in other settings and populations.

Developing HRQoL measures for young children in LMICs presents challenges such as poor literacy levels that hinder accurate reporting of HRQoL and the subjectivity of proxy reports, which reflect caregiver perceptions rather than the child’s true HRQoL. This item bank offers options for overcoming these limitations in future measure development. A new HRQoL measure will allow clinicians and researchers to assess better and compare the HRQoL of children throughout their health journey and in different settings.

## Conclusion

This development process incorporated input from potential end-users, the target population, and published literature. The findings from this study provide the first disease-specific item bank tailored to young children affected by respiratory illnesses in an LMIC, suitable for use in public health settings. Further assessment of the measure is required to ensure its validity and reliability. Continued research and collaboration will enhance the validity and reliability across various LMIC contexts.

## Electronic supplementary material

Below is the link to the electronic supplementary material.

Supplementary Material 1

Supplementary Material 2

Supplementary Material 3

## Data Availability

The Health Research Ethics Committee does not allow us to share (deidentified) data without a data-sharing agreement. The data used and/or analysed within this study are available from the corresponding author or upon reasonable request under a data-sharing agreement.
