# Supplementary material for: An item bank to measure health-related quality of life among young children (0-5-years-old) affected by respiratory illnesses – expert stakeholders and end-users from the Western Cape, South Africa
Source: Health Qual Life Outcomes. 2024 Oct 29;22:95. doi: 10.1186/s12955-024-02308-0 (PMC11523652; doi:10.1186/s12955-024-02308-0)
Supplement: Supplementary file 1 — Supplementary Material 1 [file 12955_2024_2308_MOESM1_ESM.docx]

**Additional file 1: Cohort 1 and Cohort 2 stages of item development and modifications**

| **COHORT 1: 0-2-YEAR-OLDS** | | | | |
| --- | --- | --- | --- | --- |
| **#** | **Items as initially drafted** | **Items after the delphi study** | **Items after cognitive interview** | **Final items after stakeholder consultations** |
| **DOMAIN 1: PHYSICAL HEALTH** | | | | |
| 1 | My child was able to keep up when playing with his/her friends/siblings | My child was able to keep up when playing with his/her friends/siblings | My child was able to keep up when playing with his/her friends/siblings | My child was able to keep up when playing with his/her friends/siblings. |
| 2 | My child had fun playing | My child had fun playing | My child had fun playing | My child had fun playing |
| 3 | My child had trouble sleeping because of his/her symptoms (incl. cough, pain, fever etc.) | My child had trouble sleeping because of his/her symptoms (incl. cough, pain, fever etc.) | My child had trouble sleeping because of his/her symptoms (incl. cough, pain, fever etc.) | My child had trouble sleeping because of his/her symptoms (incl. cough pain, fever etc.) |
| 4 | My child felt tired | My child felt tired | My child felt tired | My child felt tired |
| 5 | My child refused to eat | My child refused to eat | My child refused to eat | My child refused to eat |
| 6 | My child was full of energy | My child was full of energy | My child was full of energy | My child was full of energy |
| 7 | My child felt strong | ~~My child felt strong~~^a^ | ~~My child felt strong~~^a^ | ~~My child felt strong~~^a^ |
| 8 | My child did not feel like playing | My child did not feel like playing | My child did not feel like playing | My child did not feel like playing |
| 9 | My child was active | My child was active | My child was active | My child was active |
| 10 | My child felt sick | My child felt sick | My child felt sick | My child felt sick |
| 11 | My child had less fun than usual | My child had less fun than usual | My child had less fun than usual | My child had less fun than usual |
| 12 | My child's health is good | My child's health is good | My child's health is good | My child's health is good |
| 13 | My child had pain | My child had pain | My child had pain | My child had pain |
| 14 | My child had trouble gaining weight | My child had trouble gaining weight | My child had trouble gaining weight | My child had trouble gaining weight |
| 15 | My child's cough woke him/her up | My child's cough woke him/her up | My child's cough woke him/her up | My child's cough woke him/her up |
| 16 | My child had feeding problems | My child had feeding problems | My child had feeding problems | My child had feeding problems |
| 17 |  |  |  | My child is growing well (incl. weight and height) |
| 18 |  |  |  | My child has difficulty eating a serving of food (e.g. milk, food) |
| 19 |  |  |  | My child eats an adequate amount of food/milk to sustain his/her growth at an age-appropriate level) |
| 20 |  |  |  | My child struggles to gain weight |
| 21 |  |  |  | My child is shorter (height) than other children his/her age. |
| 22 |  |  |  | My child has limited appetite (e.g. milk, food) |
| 23 |  |  |  | My child eats well (maintains his/her growth at an age-appropriate level) |
| 24 |  |  |  | My child is growing well (incl. weight and height) |
| 25 |  |  |  | My child gets tired easily when he/she is playing |
| 26 |  |  |  | My child gets tired easily when he/she is rolling/crawling |
| 27 |  |  |  | My child has to take tablets/syrup/puffs or other medication daily |
| 28 |  |  |  | My child does not like taking his/her medication |
| 29 |  |  |  | My child does not like the taste of his/her medication, so I have to hide the taste of the medication to make it taste better. |
| 30 |  |  |  | My child had to go to the clinic. |
| 31 |  |  |  | My child has been admitted to the hospital |
| 32 |  |  |  | My child’s medication makes him/her feel sick |
| 33 |  |  |  | My child refuses to take his/her medication |
| **DOMAIN 2: EMOTIONAL HEALTH** | | | | |
| 1 | My child was crying when he/she is sick | My child was crying when he/she is sick | My child was crying when he/she is sick | My child is crying more often than usual* |
| 2 | My child felt as if he/she was being punished | ~~My child felt as if he/she was being punished~~^a^ | ~~My child felt as if he/she was being punished~~^a^ | ~~My child felt as if he/she was being punished~~^a^ |
| 3 | My child thinks that his/her friends with make fun of him/her | ~~My child thinks that his/her friends with make fun of him/her~~^a^ | ~~My child thinks that his/her friends with make fun of him/her~~^a^ | ~~My child thinks that his/her friends with make fun of him/her~~^a^ |
| 4 | My child thinks his/her friends with make fun of him/her because of the way he/she looks | ~~My child thinks his/her friends with make fun of him/her because of the way he/she looks~~^a^ | ~~My child thinks his/her friends with make fun of him/her because of the way he/she looks~~^a^ | ~~My child thinks his/her friends with make fun of him/her because of the way he/she looks~~^a^ |
| 5 | My child felt scared | ~~My child felt scared~~^a^ | ~~My child felt scared~~^a^ | ~~My child felt scared~~^a^ |
| 6 | My child was unhappy | My child was unhappy | My child was unhappy | My child was more unhappy than usual* |
| 7 | My child was happy | My child was happy | My child was happy | My child was happier than usual* |
| 8 | My child was anxious | My child was anxious | ~~My child was anxious~~^b^ | ~~My child was anxious~~^b^ |
| 9 | My child was angry | My child was angry | My child was angry | My child was more angry than usual* |
| 10 | My child was sad | My child was sad | My child was sad | My child was more sad than usual* |
| 11 | My child felt lonely | ~~My child felt lonely~~^a^ | ~~My child felt lonely~~^a^ | ~~My child felt lonely~~^a^ |
| 12 | My child felt excited | My child felt excited | My child felt excited | ~~My child felt excited~~^c^ |
| 13 | My child was irritable | My child was irritable | My child was irritable | My child was more irritable than usual* |
| 14 | My child was short-tempered | ~~My child was short-tempered~~^a^ | ~~My child was short-tempered~~^a^ | ~~My child was short-tempered~~^a^ |
| 15 | My child was fussy | ~~My child was fussy~~^a^ | ~~My child was fussy~~^a^ | ~~My child was fussy~~^a^ |
| 16 | My child's cry was inconsolable | My child's cry was inconsolable | My child’s cry was ~~inconsolable~~  broken-hearted | My child's cry was more broken-hearted than usual* |
| 17 |  |  | My child was worried | ~~My child was worried~~^c^ |
| **DOMAIN 3: BEHAVIOURAL EXPRESSIONS** | | | | |
| 1 | My child felt uncomfortable when he/she was sick | My child felt uncomfortable when he/she was sick | My child felt uncomfortable when he/she was sick | My child felt more uncomfortable than usual* |
| 2 | My child was nagging more when he/she was sick | My child was nagging more when he/she was sick | My child was nagging more when he/she was sick | My child was nagging more than usual* |
| 3 | My child was shy | My child was shy | ~~My child was shy~~^b^ | ~~My child was shy~~^b^ |
| 4 | My child felt proud of himself/herself | My child felt proud of himself/herself | ~~My child felt proud of himself/herself~~^b^ | ~~My child felt proud of himself/herself~~^b^ |
| 5 | My child felt that he/she was physically different to other children his/her age | ~~My child felt that he/she was physically different to other children his/her age~~^a^ | ~~My child felt that he/she was physically different to other children his/her age~~^a^ | ~~My child felt that he/she was physically different to other children his/her age~~^a^ |
| 6 | My child felt jealous about the way other girls and boys look | ~~My child felt jealous about the way other girls and boys look~~^a^ | ~~My child felt jealous about the way other girls and boys look~~^a^ | ~~My child felt jealous about the way other girls and boys look~~^a^ |
| 7 | My child is more withdrawn | My child is more withdrawn | My child is more withdrawn | My child was more ~~withdrawn~~ distant than usual* |
| 8 | My child is moody | My child is moody | My child is moody | My child was moodier than usual* |
| 9 | My child was defiant | My child was defiant | My child was ~~defiant~~ naughty | My child was naughtier than usual* |
| 10 | My child was restless | My child was restless | My child was restless | My child was more restless than usual* |
| 11 | My child was fidgety | My child was fidgety | My child was ~~fidgety~~ busy | My child was busier (fidgety) than usual* |
| 12 | My child was needy | My child was needy | My child was needy | My child ~~was needy~~ needed more attention than usual* |
| 13 | My child was clingy | My child was clingy | My child was ~~clingy~~ did not want to let me go | My child did not want to let me go more often than usual* |
| 14 | My child had nightmares | My child had nightmares | My child had nightmares | My child had more nightmares than usual* |
| 15 | My child started wetting his/her pants | ~~My child started wetting his/her pants~~^a^ | ~~My child started wetting his/her pants~~^a^ | ~~My child started wetting his/her pants~~^a^ |
| 16 | My child started wetting his/her bed | ~~My child started wetting his/her bed~~^a^ | ~~My child started wetting his/her bed~~^a^ | ~~My child started wetting his/her bed~~^a^ |
| 17 |  |  |  | My child was more uncooperative than usual |
| 18 |  |  |  | My child was more attached to us as parents than usual |
| 19 |  |  |  | My child was more attached to his/her grandmother, grandfather, aunty, uncle, cousins etc. than usual |
| 20 |  |  |  | My child was more easily comforted when he/she is upset or crying by contact with the parent than usual |
| 21 |  |  |  | My child was more demanding and impatient with his/her parents than usual |
| 22 |  |  |  | My child cried more than usual to get our attention |
| 23 |  |  |  | My child separated from us as parents more easily than usual |
| 24 |  |  |  | My child was more anxious when he/she was surrounded by unfamiliar people (e.g. clinic and hospital staff) than usual |
| **DOMAIN 4: SOCIAL WELL-BEING** | | | | |
| 1 | My child's friends left him/her out when they did things (e.g., playing) together | My child's friends left him/her out when they did things (e.g., playing) together | ~~My child's friends left him/her out when they did things (e.g., playing) together~~^b^ | ~~My child's friends left him/her out when they did things (e.g., playing) together~~^b^ |
| 2 | My child's brothers, sisters or cousins left him/her out when they did things (e.g., playing) together | My child's brothers, sisters or cousins left him/her out when they did things (e.g., playing) together | My child's brothers, sisters or cousins left him/her out when they did things (e.g., playing) together | ~~My child's brothers, sisters or cousins left him/her out when they did things (e.g., playing) together~~^c^ |
| 3 | My child was felt confident with other children | My child was felt confident with other children | ~~My child was felt confident with other children~~^b^ | ~~My child was felt confident with other children~~^b^ |
| 4 | My child felt scared around other children | My child felt scared around other children | My child felt scared around other children | My child felt scared around other children |
| 5 | My child's friend helped him/her | ~~My child's friend helped him/her~~^a^ | ~~My child's friend helped him/her~~^a^ | ~~My child's friend helped him/her~~^a^ |
| 6 | My child helped her friends | ~~My child helped her friends~~^a^ | ~~My child helped her friends~~^a^ | ~~My child helped her friends~~^a^ |
| 7 | My child got on well with his/her brothers, sisters, cousins | My child got on well with his/her brothers, sisters, cousins | My child got on well with his/her brothers, sisters, cousins | My child generally gets on well with his/her brothers, sisters, cousins |
| 8 | My child got on well with his/her friends | My child got on well with his/her friends | My child got on well with his/her friends | ~~My child got on well with his/her friends~~^c^ |
| 9 | My child had difficulty getting along with others | My child had difficulty getting along with others | ~~My child had difficulty getting along with others~~^b^ | ~~My child had difficulty getting along with others~~^b^ |
| 10 | My child's illness/disease caused stress in the family | My child's illness/disease caused stress in the family | My child's illness/disease caused stress in the family | My child's illness/disease caused stress in the family |
| 11 | My child was liked by other children | My child was liked by other children | ~~My child was liked by other children~~^b^ | ~~My child was liked by other children~~^b^ |
| 12 |  |  |  | My child had difficulty getting along with other children |
| 13 |  |  |  | My child can play with his/her brothers, sisters, and cousins |
| 14 |  |  |  | My child enjoys the company of his/her friends |
| 15 |  |  |  | My child shares and takes turns with his/her friends |
| 16 |  |  |  | My child responds to the voice of an adult and smiles |
| 17 |  |  |  | My child stops crying at the sound of his/her parents voice |
| 18 |  |  |  | My child spontaneously smiles at sights or sounds that are pleasurable |
| **DOMAIN 5: FEELING LOVE** | | | | |
| 1 | My community came over to helped care for my child when he/she was sick | My community came over to helped care for my child when he/she was sick | My community came over to help care for my child when he/she was sick | My ~~community~~ child’s support network came over to help care ~~for him/her when he/she was sick*~~ |
| 2 | My child got on well with his/her family | My child got on well with his/her family | My child got on well with his/her family | My child generally gets on well with his/her ~~family~~ support network* |
| 3 | My child felt unloved by his/her family | ~~My child felt unloved by his/her family~~^a^ | ~~My child felt unloved by his/her family~~^a^ | ~~My child felt unloved by his/her family~~^a^ |
| 4 | My child felt loved by his/her family (including brothers, sisters, cousins, mother, father, extended family) | My child felt loved by his/her family (including brothers, sisters, cousins, mother, father, extended family) | My child felt loved by his/her family (including brothers, sisters, cousins, mother, father, extended family) | My child felt loved by his/her ~~family~~ support network (including brothers, sisters, cousins, mother, father, extended family etc.)* |
| 5 | My family comforted my child when he/she was crying, not feeling sick. | My family comforted my child when he/she was crying, not feeling sick. | My family comforted my child when he/she was crying, not feeling sick. | My child’s ~~family~~ support network comforted him/her when he/she was crying/unhappy/sick* |
| 6 | My family helped care for my child when he/she was feeling sick. | My family helped care for my child when he/she was feeling sick. | My family helped care for my child when he/she was feeling sick. | My child’s ~~family~~ support network helped care for him/her* |
| 7 | My child felt loved by his/her community/neighbours. | My child felt loved by his/her community/neighbours. | My child felt loved by his/her community/neighbours. | My child felt loved by his/her ~~community/neighbours~~ support network* |
| 8 |  |  | My family did not support my child when he/she was feeling sick | My ~~family~~ child’s support network did not support him/her* |
| **DOMAIN 6: EARLY DEVELOPMENT** | | | | |
| 1 | My child communicates in an age-appropriate way with his/her friends (babbling, smiling, touching) | My child communicates in an age-appropriate way with his/her friends (babbling, smiling, touching, talking) | My child communicates in an age-appropriate way with his/her friends (babbling, smiling, touching, talking) | My child communicates in an age-appropriate way with his/her friends (babbling, smiling, touching, talking) |
| 2 | My child can talk in complete sentences | My child can talk in complete sentences | ~~My child can talk in complete sentences~~^b^ | ~~My child can talk in complete sentences~~^b^ |
| 3 | My child can follow simple commands | ~~My child can follow simple commands~~^a^ | ~~My child can follow simple commands~~^a^ | ~~My child can follow simple commands~~^a^ |
| 4 | My child was not able to things other children his/her age can do. | My child was not able to do things other children his/her age can do. | My child was not able to do things other children his/her age can do. | My child was not able to do things other children his/her age can do. |
| 5 | My child can imitate others | My child can imitate others | My child can ~~imitate~~ copy others | My child can ~~imitate~~ copy others |
| 6 | My child is behind other child his/her age in day care | My child is behind other child his/her age in day care | My child is behind other child his/her age in day care | My child is behind other child his/her age in day care |
| 7 | My child communicates in an age-appropriate way with his/her family | My child communicates in an age-appropriate way with his/her family | My child communicates in an age-appropriate way with his/her family | My child communicates in an age-appropriate way with his/her family |
| 8 | My child can keep up with other children his/her age at day care/creche | My child can keep up with other children his/her age at day care/creche | My child can keep up with other children his/her age at day care/creche | My child can keep up with other children his/her age at day care/creche |
| 9 | My child needed extra support at daycare/creche | My child needed extra support at daycare/creche | My child needed extra support at daycare/creche | ~~My child needed extra support at daycare/creche~~^c^ |
| 10 | My child enjoyed day care/ creche | My child enjoyed day care/ creche | My child enjoyed day care/ creche | My child enjoyed day care/ creche |
| 11 |  |  |  | My child is learning to communicate or learning language in age age-appropriate way (e.g. smiling, making noises, repeating sounds, responding to his/her name etc.) |
| 12 |  |  |  | My child can rollover |
| 13 |  |  |  | my children can bring an object he/she is holding to his/her mouth |
| 14 |  |  |  | My child can sit up (with pillows to prop him/her up) |
| 15 |  |  |  | My child can crawl. |
| 16 |  |  |  | my child can scribble with markers or crayons. |
| 17 |  |  |  | my child can jump around. |
| 18 |  |  |  | My child can pull toys behind him/her while walking |
| 19 |  |  |  | My child can express himself/herself (including making cooing noises, smiling, laughing etc.) |
| 20 |  |  |  | My child enjoys the company of his/her friends |
| 21 |  |  |  | My child participates in age-appropriate play (e.g., grasping blocks, rattles, teddy bear, stuffed animals) |
| 22 |  |  |  | My child enjoys playing alone |
| 23 |  |  |  | My child shows interest in stories |
| **DOMAIN 7: ROUTINE** | | | | |
| 1 | My child was not eating enough because he/she was not feeling well. | My child was not eating enough because he/she was not feeling well. | My child was not eating enough because he/she was not feeling well. | My child was not eating enough because he/she was not feeling well. |
| 2 | My family had to change our routine when my child was sick | My family had to change our routine when my child was sick | My family had to change our routine when my child was sick | ~~My family had to change our routine when my child was sick~~^c^ |
| 3 | My child had difficulty staying asleep because of his/her symptoms | My child had difficulty staying asleep because of his/her symptoms | My child had difficulty staying asleep because of his/her symptoms | My child slept more often ~~had difficulty staying asleep~~ because of his/her symptoms* |
| 4 | My child's sleeping patterns were irregular/changed/disturbed | My child's sleeping patterns were irregular/changed/disturbed | My child's sleeping patterns were irregular/changed/disturbed | My child's sleeping patterns were irregular/changed/disturbed |
| 5 | My child missed out on creche/day care because he/she was sick | My child missed out on creche/day care because he/she was sick | My child missed out on creche/day care because he/she was sick | ~~My child missed out on creche/day care because he/she was sick~~^c^ |
| 6 | My child stuck to his/her sleeping routine | My child stuck to his/her sleeping routine | My child stuck to his/her sleeping routine | My child slept more than usual ~~stuck to his/her sleeping routine*~~ |
| 7 | My child stuck to his/her eating routine | My child stuck to his/her eating routine | My child stuck to his/her eating routine | My child stuck to his/her eating routine |
| 8 | My child’s normal routine was changed when he/she was sick | My child’s normal routine was changed when he/she was sick | My child’s normal routine was changed when he/she was sick | ~~My child’s normal routine was changed when he/she was sick~~ My child follows a routine* |
| 9 |  |  |  | My child is cared for by a babysitter, family or community when he/she is sick and cannot go to creche/daycare |
| 10 |  |  |  | My child wakes up more often than usual when he/she is sick |

1. Item strikethrough^a.^ = Items removed in the Delphi study

2. Item strikethrough^b^ = Items removed in Cognitive interviews

3. Item strikethrough^c^ = Items removed in Stakeholder consultations

4. Item with Asterix = Item further modified by stakeholders

| **COHORT 2: 3-5-YEAR-OLDS** | | | | |
| --- | --- | --- | --- | --- |
| **#** | **Item as initially drafted** | **Item after the delphi study** | **Item after cognitive interview** | **Final item after stakeholder consultations** |
| **DOMAIN 1: PHYSICAL HEALTH** | | | | |
| 1 | My child was able to keep up when playing with his/her friends/siblings | My child was able to keep up when playing with his/her friends/siblings | My child was able to keep up when playing with his/her friends/siblings | My child was able to keep up when playing with his/her friends/siblings |
| 2 | My child had fun playing | My child had fun playing | My child had fun playing | My child had fun playing |
| 3 | My child had trouble sleeping because of his/her symptoms (incl. cough, pain, fever etc.) | My child had trouble sleeping because of his/her symptoms (incl. cough, pain, fever etc.) | My child had trouble sleeping because of his/her symptoms (incl. cough, pain, fever etc.) | My child had trouble sleeping because of his/her symptoms (incl. cough, pain, fever etc.) |
| 4 | My child felt tired | My child felt tired | My child felt tired | My child felt tired |
| 5 | My child refused to eat | My child refused to eat | My child refused to eat | My child refused to eat |
| 6 | My child was full of energy | My child was full of energy | My child was full of energy | My child was full of energy |
| 7 | My child felt strong | My child felt strong | ~~My child felt strong~~^b^ | ~~My child felt strong~~^b^ |
| 8 | My child did not feel like playing | My child did not feel like playing | My child did not feel like playing | My child did not feel like playing |
| 9 | My child was active | My child was active | My child was active | My child was active |
| 10 | My child felt sick | My child felt sick | My child felt sick | My child felt sick |
| 11 | My child had less fun than usual | My child had less fun than usual | My child had less fun than usual | My child had less fun than usual |
| 12 | My child's health is good | My child's health is good | My child's health is good | My child's health is good |
| 13 | My child had pain | My child had pain | My child had pain | My child had pain |
| 14 | My child had trouble gaining weight | My child had trouble gaining weight | My child had trouble gaining weight | My child had trouble gaining weight |
| 15 | My child's cough woke him/her up | My child's cough woke him/her up | My child's cough woke him/her up | My child's cough woke him/her up |
| 16 | My child had feeding problems | My child had feeding problems | My child had feeding problems | My child had feeding problems |
| 17 |  |  |  | My child moves freely and with confidence in a range of ways |
| 18 |  |  |  | My child jumps up and down landing on both his/her feet |
| 19 |  |  |  | My child is growing well (incl. weight and height) |
| 20 |  |  |  | My child has difficulty eating a serving of food |
| 21 |  |  |  | My child eats an adequate amount of food to sustain his/her growth at an age-appropriate level) |
| 22 |  |  |  | My child struggles to gain weight |
| 23 |  |  |  | My child is shorter (height) than other children his/her age. |
| 24 |  |  |  | My child had to take tablets/puffs/syrup or other medication daily |
| 25 |  |  |  | My child does not like taking his/her medication |
| 26 |  |  |  | My child does not like the taste of his/her medication, so I have to hide the taste of the medication to make it taste better |
| 27 |  |  |  | My child has been admitted to the hospital |
| 28 |  |  |  | My child’s medication makes him/her feel sick |
| 29 |  |  |  | My child refuses to take his/her medication |
| 30 |  |  |  | My child gets tired easily when he/she is playing |
| 31 |  |  |  | My child gets tired easily when he/she is eating |
| 32 |  |  |  | My child gets tired easily when he/she is walking |
| **DOMAIN 2: EMOTIONAL HEALTH** | | | | |
| 1 | My child was crying when he/she is sick | My child was crying when he/she is sick | My child was crying when he/she is sick | My child was crying when he/she is sick |
| 2 | My child felt as if he/she was being punished | My child felt as if he/she was being punished | ~~My child felt as if he/she was being punished~~^b^ | ~~My child felt as if he/she was being punished~~^b^ |
| 3 | My child thinks that his/her friends with make fun of him/her | ~~My child thinks that his/her friends with make fun of him/her~~^a^ | ~~My child thinks that his/her friends with make fun of him/her~~^a^ | ~~My child thinks that his/her friends with make fun of him/her~~^a^ |
| 4 | My child thinks his/her friends with make fun of him/her because of the way he/she looks | ~~My child thinks his/her friends with make fun of him/her because of the way he/she looks~~^a^ | ~~My child thinks his/her friends with make fun of him/her because of the way he/she looks~~^a^ | ~~My child thinks his/her friends with make fun of him/her because of the way he/she looks~~^a^ |
| 5 | My child felt scared | My child felt scared | My child felt scared | My child felt scared |
| 6 | My child was unhappy | My child was unhappy | My child was unhappy | My child was more unhappy than usual* |
| 7 | My child was happy | My child was happy | My child was happy | My child was happier than usual* |
| 8 | My child was anxious | My child was anxious | My child was anxious | My child was more anxious when he/she had to go to the doctor, clinic, hospital* |
| 9 | My child was angry | My child was angry | My child was angry | My child was angry |
| 10 | My child was sad | My child was sad | My child was sad | My child was more sad than usual* |
| 11 | My child felt lonely | My child felt lonely | My child felt lonely | My child felt lonelier than usual* |
| 12 | My child felt excited | My child felt excited | My child felt excited | My child felt excited |
| 13 | My child was irritable | My child was irritable | My child was irritable | My child was more irritable than usual* |
| 14 | My child was short-tempered | My child was short-tempered | ~~My child was short-tempered~~^b^ | ~~My child was short-tempered~~^c^ |
| 15 | My child was fussy | My child was fussy | My child was ~~fussy~~ complaining | My child was ~~fussy~~ complaining more than usual* |
| 16 | My child's cry was inconsolable | My child's cry was inconsolable | My child's cry was ~~inconsolable~~ broken-hearted | My child's cry was more broken-hearted ~~inconsolable~~ than usual* |
| 17 |  |  | My child was worried | ~~My child was worried~~^c^ |
| **DOMAIN 3: PSYCHOLOGICAL HEALTH** | | | | |
| 1 | My child felt uncomfortable when he/she was sick | My child felt uncomfortable when he/she was sick | My child felt uncomfortable when he/she was sick | My child felt more uncomfortable than usual |
| 2 | My child was nagging more when he/she was sick | My child was nagging more when he/she was sick | My child was nagging more when he/she was sick | My child was nagging more than usual* |
| 3 | My child was shy | My child was shy | ~~My child was shy~~^b^ | ~~My child was shy~~^b^ |
| 4 | My child felt proud of himself/herself | My child felt proud of himself/herself | ~~My child felt proud of himself/herself~~^b^ | ~~My child felt proud of himself/herself~~^b^ |
| 5 | My child felt that he/she was physically different to other children his/her age | ~~My child felt that he/she was physically different to other children his/her age~~^a^ | ~~My child felt that he/she was physically different to other children his/her age~~^a^ | ~~My child felt that he/she was physically different to other children his/her age~~^a^ |
| 6 | My child felt jealous about the way other girls and boys look | ~~My child felt jealous about the way other girls and boys look~~^a^ | ~~My child felt jealous about the way other girls and boys look~~^a^ | ~~My child felt jealous about the way other girls and boys look~~^a^ |
| 7 | My child is more withdrawn | My child is more withdrawn | My child is more quiet ~~withdrawn~~ | My child was more quiet ~~withdrawn~~ than usual* |
| 8 | My child is moody | My child is moody | My child is moody | My child was moodier than usual* |
| 9 | My child was defiant | My child was defiant | My child was naughty ~~defiant~~ | My child was naughtier than usual* |
| 10 | My child was restless | My child was restless | My child was restless | My child was more restless than usual* |
| 11 | My child was fidgety | My child was fidgety | My child was ~~fidgety~~ busy | My child was busier (fidgety) than usual* |
| 12 | My child was needy | My child was needy | My child was needy | My child ~~was needy~~ needed more attention than usual* |
| 13 | My child was clingy | My child was clingy | My child was ~~clingy~~ held onto me | My child was ~~clingy~~ held onto me |
| 14 | My child had nightmares | My child had nightmares | My child had nightmares | My child had more nightmares than usual* |
| 15 | My child started wetting his/her pants | My child started wetting his/her pants | ~~My child started wetting his/her pants~~^b^ | ~~My child started wetting his/her pants~~^b^ |
| 16 | My child started wetting his/her bed | My child started wetting his/her bed | ~~My child started wetting his/her bed~~^b^ | ~~My child started wetting his/her bed~~^b^ |
| 17 |  |  |  | My child was more short-tempered than usual |
| 18 |  |  |  | My child was more uncooperative than usual |
| 19 |  |  |  | My child could identify things he/she fears |
| 20 |  |  |  | My child was worried about being away from his/her family |
| 21 |  |  |  | My child was more scared of sleeping on his/her own than usual |
| 22 |  |  |  | My child was more scared when he/she had to go the doctor/hospital than usual |
| 23 |  |  |  | My child was more confident taking on new things than usual |
| 24 |  |  |  | My child was happy with who he/she is |
| 25 |  |  |  | My child was more attached to us as parents than usual |
| 26 |  |  |  | My child was more attached to his/her grandmother, grandfather, aunty, uncle, cousins etc. than usual |
| 27 |  |  |  | My child was more easily comforted when he/she is upset or crying by contact with the parent than usual |
| 28 |  |  |  | My child was more demanding and impatient with his/her parents than usual |
| 29 |  |  |  | My child cried more than usual to get our attention |
| 30 |  |  |  | My child separated from us as parents more easily than usual |
|  |  |  |  | My child was more anxious when he/she was surrounded by unfamiliar people (e.g. clinic and hospital staff) than usual |
| 31 |  |  |  | My child was more defiant than usual |
| **DOMAIN 4: SOCIAL WELL-BEING** | | | | |
| 1 | My child's friends left him/her out when they did things (e.g., playing) together | My child's friends left him/her out when they did things (e.g., playing) together | ~~My child's friends left him/her out when they did things (e.g., playing) together~~^b^ | ~~My child's friends left him/her out when they did things (e.g., playing) together~~^b^ |
| 2 | My child's brothers, sisters or cousins left him/her out when they did things (e.g., playing) together | My child's brothers, sisters or cousins left him/her out when they did things (e.g., playing) together | My child's brothers, sisters or cousins left him/her out when they did things (e.g., playing) together | My child's brothers, sisters or cousins left him/her out when they did things (e.g., playing) together |
| 3 | My child was felt confident with other children | My child was felt confident with other children | ~~My child was felt confident with other children~~^b^ | ~~My child was felt confident with other children~~^b^ |
| 4 | My child felt scared around other children | My child felt scared around other children | My child felt scared around other children | My child felt scared around other children |
| 5 | My child's friend helped him/her | My child's friend helped him/her | ~~My child's friend helped him/her~~^b^ | ~~My child's friend helped him/her~~^b^ |
| 6 | My child helped her friends | My child helped her friends | ~~My child helped her friends~~^b^ | ~~My child helped her friends~~^b^ |
| 7 | My child got on well with his/her brothers, sisters, cousins | My child got on well with his/her brothers, sisters, cousins | My child got on well with his/her brothers, sisters, cousins | My child generally gets ~~got~~ on well with his/her brothers, sisters, cousins* |
| 8 | My child got on well with his/her friends | My child got on well with his/her friends | My child got on well with his/her friends | My child generally gets ~~got~~ on well with his/her friends* |
| 9 | My child had difficulty getting along with others | My child had difficulty getting along with others | My child had difficulty getting along with others | My child generally has ~~had~~ difficulty getting along with others* |
| 10 | My child's illness/disease caused stress in the family | My child's illness/disease caused stress in the family | My child's illness/disease caused stress in the family | My child's illness/disease caused stress in the family |
| 11 | My child was liked by other children | My child was liked by other children | ~~My child was liked by other children~~^b^ | ~~My child was liked by other children~~^b^ |
| 12 |  |  |  | My child can play with his/her brothers, sisters and cousins |
| 13 |  |  |  | My child enjoys the company of his/her friends |
| 14 |  |  |  | My child shares and takes turns with his/her friends |
| 15 |  |  |  | My child can play alongside other children |
| 16 |  |  |  | My child shows concern for the needs of other children |
| 17 |  |  |  | My child is starting to understand the concept of sharing and taking turns with others |
| **DOMAIN 5: FEELING LOVE** | | | | |
| 1 | My community came over to helped care for my child when he/she was sick | My community came over to helped care for my child when he/she was sick | My community came over to helped care for my child when he/she was sick | My ~~community~~ child’s support network came over to help care for him/her* |
| 2 | My child got on well with his/her family | My child got on well with his/her family | My child got on well with his/her family | ~~My child got on well with his/her family~~^c^ |
| 3 | My child felt unloved by his/her family | My child felt unloved by his/her family | ~~My child felt unloved by his/her family~~^b^ | ~~My child felt unloved by his/her family~~^b^ |
| 4 | My child felt loved by his/her family (including brothers, sisters, cousins, mother, father, extended family) | My child felt loved by his/her family (including brothers, sisters, cousins, mother, father, extended family) | My child felt loved by his/her family (including brothers, sisters, cousins, mother, father, extended family) | My child felt loved by his/her ~~family~~ support network (including brothers, sisters, cousins, mother, father, extended family etc.)* |
| 5 | My family comforted my child when he/she was crying, not feeling sick. | My family comforted my child when he/she was crying, not feeling sick. | My family comforted my child when he/she was crying, not feeling sick. | My ~~family~~ child’s support network comforted him/her when he/she was crying/unhappy/sick* |
| 6 | My family helped care for my child when he/she was feeling sick. | My family helped care for my child when he/she was feeling sick. | My family helped care for my child when he/she was feeling sick. | My ~~family~~ child’s support network helped care for him/her* |
| 7 | My child felt loved by his/her community/neighbours. | My child felt loved by his/her community/neighbours. | My child felt loved by his/her community/neighbours. | My child felt loved by his/her ~~family/community/people~~ support network* |
| 8 |  |  | My family did not support my child when he/she was feeling sick | My ~~family~~ child’s support network did not support him/her* |
| **DOMAIN 6: PRESCHOOL READINESS** | | | | |
| 1 | My child communicates in an age-appropriate way with his/her friends (babbling, smiling, touching) | My child communicates in an age-appropriate way with his/her friends (babbling, smiling, touching) | My child communicates in an age-appropriate way with his/her friends (babbling, smiling, touching, or talking) | My child can communicate ~~in an age-appropriate way~~ his/her wishes with his/her friends (smiling, or talking)* |
| 2 | My child can talk in complete sentences | My child can talk in complete sentences | ~~My child can talk in complete sentences~~^b^ | ~~My child can talk in complete sentences~~^b^ |
| 3 | My child can follow simple commands | My child can follow simple commands | My child can follow simple commands | My child can follow simple commands |
| 4 | My child was not able to do things other children his/her age can do | My child was not able to do things other children his/her age can do | My child was not able to do things other children his/her age can do | My child was not able to do things other children his/her age can do |
| 5 | My child can imitate others | My child can imitate others | My child can ~~imitate~~ copy others | ~~My child can imitate copy others~~^c^ |
| 6 | My child is behind other child his/her age in day care | My child is behind other child his/her age in day care | My child is behind other child his/her age in day care | My child is behind other child his/her age in day care |
| 7 | My child communicates in an age-appropriate way with his/her family | My child communicates in an age-appropriate way with his/her family | My child communicates in an age-appropriate way with his/her family | My child communicates in an age-appropriate way with his/her family |
| 8 | My child can keep up with other children his/her age at day care/creche | My child can keep up with other children his/her age at day care/creche | My child can keep up with other children his/her age at day care/creche | My child can keep up with other children his/her age at day care/creche |
| 9 | My child needed extra support at daycare/creche | My child needed extra support at daycare/creche | My child needed extra support at daycare/creche | ~~My child needed extra support at daycare/creche~~^c^ |
| 10 | My child enjoyed day care/ creche | My child enjoyed day care/ creche | My child enjoyed day care/ creche | My child enjoyed day care/ creche/preschool |
| 11 |  |  |  | My child enjoys the company of his/her friends |
| 12 |  |  |  | My child participates in age-appropriate play (e.g. climbing, swinging, drawing, jumping) |
| 13 |  |  |  | My child enjoys playing alone |
| 14 |  |  |  | My child takes part in pretend play (e.g. being superheroes, shopping, tea parties etc.) |
| 15 |  |  |  | My child assists with self-care (dressing, washing etc.) |
| 16 |  |  |  | My child can brush his/her teeth |
| 17 |  |  |  | My child chooses his/her own clothes |
| 18 |  |  |  | My child can answer basic questions |
| 19 |  |  |  | My child listens to detail in a story and can retell it |
| 20 |  |  |  | My child asks ‘who’ and ‘when’ questions |
| 21 |  |  |  | My child can recognise his/her own name |
| 22 |  |  |  | My child listens and responds appropriately |
| 23 |  |  |  | My child communicates in an age-appropriate way with his/her friends |
| 24 |  |  |  | My child can build towers with blocks |
| **DOMAIN 7: ROUTINE** | | | | |
| 1 | My child was not eating enough because he/she was not feeling well. | My child was not eating enough because he/she was not feeling well. | My child was not eating enough because he/she was not feeling well. | ~~My child was not eating enough because he/she was not feeling well~~.My child has a normal eating routine* |
| 2 | My family had to change our routine when my child was sick | My family had to change our routine when my child was sick | My family had to change our routine when my child was sick | ~~My family had to change our routine when my child was sick~~^c^ |
| 3 | My child had difficulty staying asleep because of his/her symptoms | My child had difficulty staying asleep because of his/her symptoms | My child had difficulty staying asleep because of his/her symptoms | My child ~~had difficult~~y slept more often because of his/her symptoms* |
| 4 | My child's sleeping patterns were irregular/changed/disturbed | My child's sleeping patterns were irregular/changed/disturbed | My child's sleeping patterns were irregular/changed/disturbed | My child’s sleeping patterns were irregular/changed/disturbed |
| 5 | My child missed out on creche/day care because he/she was sick | My child missed out on creche/day care because he/she was sick | My child missed out on creche/day care because he/she was sick | My child missed out on creche/daycare because he/she was sick |
| 6 | My child stuck to his/her sleeping routine | My child stuck to his/her sleeping routine | My child stuck to his/her sleeping routine | My child ~~stuck to his/her sleeping routine~~ slept less than usual* |
| 7 | My child stuck to his/her eating routine | My child stuck to his/her eating routine | My child stuck to his/her eating routine | My child stuck to his/her eating routine |
| 8 | My child's normal routine was changed when he/she was sick | My child's normal routine was changed when he/she was sick | My child's normal routine was changed when he/she was sick | ~~My child's normal routine was changed when he/she was sick~~ My child follows a routine* |
| 9 |  |  |  | My child wakes up more often than usual when he/she is sick |

1. Item strikethrough^a.^ = Items removed in the Delphi study

2. Item strikethrough^b^ = Items removed in Cognitive interviews

3. Item strikethrough^c^ = Items removed in Stakeholder consultations

4. Item with Asterix = Item further modified by stakeholders
