# Supplementary material for: An item bank to measure health-related quality of life among young children (0-5-years-old) affected by respiratory illnesses – expert stakeholders and end-users from the Western Cape, South Africa
Source: Health Qual Life Outcomes. 2024 Oct 29;22:95. doi: 10.1186/s12955-024-02308-0 (PMC11523652; doi:10.1186/s12955-024-02308-0)
Supplement: Supplementary file 2 — Supplementary Material 2 [file 12955_2024_2308_MOESM2_ESM.docx]

**Additional file 2: Cognitive interview frequencies and proportions across both cohorts**

|  | **0-2-years-old (n=26)** | | | | |  | **3-5-years-old (n=13)** | | | | |
| --- | --- | --- | --- | --- | --- | --- | --- | --- | --- | --- | --- |
| **Domains** | | **# of items per domain** | **# of easy-to-understand items (%)** | **# of items removed** | **Items requiring modification** | **Domains** | | **# of items per domain** | **# of easy-to-understand items (%)** | **# of items removed** | **Items requiring modification** |
| Physical Health | | 15 | 15 (100%) | 0 (0%) | 0 (0%) | Physical Health | | 16 | 15 (94%%) | 1 (6%) | 0 (0%) |
| Emotional Health | | 10 | 7 (70%) | 2 (20%) | 1 (10%) | Emotional Health | | 14 | 10 (71%) | 2 (14%) | 2 (14%) |
| Psychological health | | 12 | 7 (53%) | 2 (17%) | 3 (25%) | Psychological health | | 14 | 12 (43%) | 4 (29%) | 4 (29%) |
| Social well-being | | 9 | 5 (56%) | 4 (44%) | 0 (0%) | Social well-being | | 11 | 6 (55%) | 5 (45%) | 0 (0%) |
| Getting love/feeling love | | 6 | 6 (100%) | 0 (0%) | 0 (0%) | Getting love/feeling love | | 7 | 6 (86%) | 1 (14%) | 0 (0%) |
| Early development | | 9 | 7 (78%) | 1 (11%) | 1 (11%) | Early development | | 10 | 8 (80%) | 1 (10%) | 1 (10%) |
| Routine | | 8 | 8 (100%) | 0 (0%) | 0 (0%) | Routine | | 8 | 8 (100%) | 0 (0%) | 0 (0%) |

Note: # = number of
