# Supplementary material for: An item bank to measure health-related quality of life among young children (0-5-years-old) affected by respiratory illnesses – expert stakeholders and end-users from the Western Cape, South Africa
Source: Health Qual Life Outcomes. 2024 Oct 29;22:95. doi: 10.1186/s12955-024-02308-0 (PMC11523652; doi:10.1186/s12955-024-02308-0)
Supplement: Supplementary file 3 — Supplementary Material 3 [file 12955_2024_2308_MOESM3_ESM.docx]

**Additional file 3: Stakeholder consultations frequencies and proportions**

| **Cohort 1: 0-2-year-olds** | | | | | **Cohort 2: 3-5-year-olds** | | | | |
| --- | --- | --- | --- | --- | --- | --- | --- | --- | --- |
| **Domains** | **Suggestion for alternative wording** | **Relevance of the domain (%)** | **# of Additional items suggested** | **# of Items modified** | **Domains** | **Suggestion for alternative wording** | **Relevance of the domain (%)** | **# of Additional items suggested** | **# of Items modified** |
| Physical health | N/A | 22 (100%) | 17 | 0 | Physical health | N/A | 22 (100%) | 16 | 0 |
| Emotional health | N/A | 18 (82%) | 0 | 0 | Emotional health | N/A | 22 (100%) | 0 | 3 |
| Psychological well-being | Behavioural expressions | 21 (95%) | 8 | 1 | Psychological well-being | N/A | 20 (91%) | 16 | 4 |
| Social well-being | N/A | 21 (95%) | 7 | 0 | Social well-being | N/A | 21 (95%) | 6 | 3 |
| Getting love/feeling love | Feeling love | 17 (77%) | 0 | 7 | Getting love/feeling love | Feeling love | 18 (82%) | 0 | 6 |
| Early development | Early childhood development | 21 (95%) | 13 | 0 | Early development | Preschool readiness | 22 (100%) | 14 | 1 |
| Routine | N/A | 21 (95%) | 2 | 2 | Routine | N/A | 20 (91%) | 1 | 4 |

Note: # = number of

Abbreviations: N/A = Not Applicable
